# Anti-CGRP Monoclonal Antibodies in Chronic Migraine: A Position Statement of the Brazilian Academy of Headache and Orofacial Pain

**DOI:** 10.7759/cureus.114201

**Published:** 2026-08-09

**Authors:** Abouch Krymchantowski, Hilton Mariano da Silva Júnior, Raimundo Pereira Silva-Néto, Carla Jevoux, Alberto Luiz C Costa, Carlos A Bordini, Carlos Augusto C De Vasconcelos, Daniela G Goncalves, Débora Grossi, Élcio Piovesan, Fabiola Dach, Flávio Rezende Filho, Francisco Pereira Júnior, Ida Fortini, Jackeline Barbosa, Jaime Olavo Marquez, José Stechman Neto, Juliana Stuginski Barbosa, Igor Bruscky, Liliane Pinto Vidor, Luciano Silva Lopes, Luiz P Queiroz, Marcelo Bigal, Marco A Arruda, Marco Bruno, Marcus T Silva, Maria Lucia Veluttini Pimentel, Mariana Beiral Hammerle, Renata Pizzo, Sandro Luiz de Andrade Matas, Tania Novaretti, Vanise Grassi, Victor Bastos

**Affiliations:** 1 Neurology, Headache Center of Rio, Rio de Janeiro, BRA; 2 Neurology, Brazilian Academy of Headache and Orofacial Pain, Rio de Janeiro, BRA; 3 Neurology, Pontifícia Universidade Católica de Campinas, Campinas, BRA; 4 Neurology, Brazilian Academy of Headache and Orofacial Pain, Campinas, BRA; 5 Neurology, Universidade Federal do Delta do Parnaíba (UFDPar), Parnaíba, BRA; 6 Neurology, Brazilian Academy of Headache and Orofacial Pain, Parnaíba, BRA; 7 Neurology, Brazilian Academy of Headache and Orofacial Pain, Valinhos, BRA; 8 Neurology, Municipal University Centre of Franca, São Paulo, BRA; 9 Neurology, Brazilian Academy of Headache and Orofacial Pain, Batatais, BRA; 10 Nutrition, Federal University of Pernambuco (UFPE), Recife, BRA; 11 Nutrition, Brazilian Academy of Headache and Orofacial Pain, Recife, BRA; 12 Dental Materials and Prosthodontics, São Paulo State University (UNESP), School of Dentistry, Araraquara, Araraquara, BRA; 13 Dental Materials and Prosthodontics, Brazilian Academy of Headache and Orofacial Pain, Araraquara, BRA; 14 Physical Therapy, Universidade de São Paulo, Ribeirão Preto, BRA; 15 Physical Therapy, Brazilian Academy of Headache and Orofacial Pain, Ribeirão Preto, BRA; 16 Neurology, Universidade Federal do Paraná, Curitiba, BRA; 17 Neurology, Brazilian Academy of Headache and Orofacial Pain, Curitiba, BRA; 18 Neurology, Universidade de São Paulo, São Paulo, BRA; 19 Neurology, Brazilian Academy of Headache and Orofacial Pain, Ribeirão Preto, BRA; 20 Neurology, Universidade Federal de São Paulo (UNIFESP), São Paulo, BRA; 21 Neurology, Brazilian Academy of Headache and Orofacial Pain, São Paulo, BRA; 22 Temporomandibular Disorders and Orofacial Pain, Brazilian Academy of Headache and Orofacial Pain, Rio de Janeiro, BRA; 23 Internal Medicine, Santa Casa de Misericórdia do Rio de Janeiro, Rio de Janeiro, BRA; 24 Internal Medicine, Brazilian Academy of Headache and Orofacial Pain, Rio de Janeiro, BRA; 25 Neurology, Federal University of Triângulo Mineiro, Uberaba, BRA; 26 Neurology, Brazilian Academy of Headache and Orofacial Pain, Uberaba, BRA; 27 Orofacial Pain, Universidade do Tuiuti, Curitiba, BRA; 28 Orofacial Pain, Brazilian Academy of Headache and Orofacial Pain, Curitiba, BRA; 29 Neurology, Bauru Orofacial Pain Group, Ribeirão Preto, BRA; 30 Neurology, Instituto Envelhecer Com Saúde, Recife, BRA; 31 Neurology, Brazilian Academy of Headache and Orofacial Pain, Recife, BRA; 32 Neurology, Associação Brasileira de Odontologia, Porto Alegre, BRA; 33 Neurology, Brazilian Academy of Headache and Orofacial Pain, Porto Alegre, BRA; 34 Biophysics and Physiology, Universidade Federal do Piauí, Teresina, BRA; 35 Biophysics and Physiology, Brazilian Academy of Headache and Orofacial Pain, Teresina, BRA; 36 Neurology, Universidade Federal de Santa Catarina, Florianópolis, BRA; 37 Neurology, Brazilian Academy of Headache and Orofacial Pain, Florianópolis, BRA; 38 Neurology, Ventus Therapeutics, New Hope, USA; 39 Neurology, Brazilian Academy of Headache and Orofacial Pain, New Hope, USA; 40 Neurology, Instituto Glia, Ribeirão Preto, BRA; 41 Neurology, Instituto Oswaldo Cruz (IOC/Fiocruz), Rio de Janeiro, BRA; 42 Neurology, Santa Casa de Misericórdia do Rio de Janeiro, Rio de Janeiro, BRA; 43 Neurology, Universidade Federal do Estado do Rio de Janeiro, Rio de Janeiro, BRA; 44 Neurology, Universidade de São Paulo em Ribeirão Preto (USP-RP), Ribeirão Preto, BRA; 45 Neurology, Universidade Federal de Ciências da Saúde de Porto Alegre, Porto Alegre, BRA

**Keywords:** anti-cgrp monoclonal antibodies, brazilian academy of headache and orofacial pain, chronic migraine, medication-overuse headache, treatment resistance

## Abstract

Background

Chronic migraine (CM) is a disabling neurological condition with high burden, common comorbidities, and a frequent profile of treatment resistance. Although there is progress in mechanism-based therapeutic approaches, such as anti-calcitonin gene-related peptide (CGRP) monoclonal antibodies (mAbs), real-world data on their clinical use and positioning in Latin America remain limited.

Methods

A cross-sectional, expert-opinion survey of 21 Brazilian headache specialists was carried out to inform a position statement of the Brazilian Academy of Headache and Orofacial Pain. The electronic survey consisted of 49 structured questions that addressed clinical experience with CM, the prevalence of comorbid medication-overuse headache (MOH), the use of anti-CGRP mAbs fremanezumab and galcanezumab as treatment options, comparative assessment, therapeutic positioning, and consensus statements. Descriptive statistics were employed, and consensus was considered as 80% agreement.

Results

Twenty-one specialists participated; 14 (66.7%) had >20 years’ experience. Misdiagnosis was common (16 (81.0%)), and 12 (57.1%) had a diagnostic delay of one year or more. The rate of treatment resistance was high (13 (61.9%) reported that over half of patients were non-responders to two or more prior preventives), and the prevalence of MOH was also high (17 (81.0%) estimated 80% coexistence or higher). The anti-CGRP mAbs were the most used therapies (fremanezumab 17 (81.0%), galcanezumab 18 (85.7%)) and were perceived by respondents as effective in reducing migraine days and improving quality of life. Safety and tolerability ratings reported by clinicians were favorable, with the most common reactions being injection-site reactions. Most clinicians did not indicate notable differences among agents, although no statistical comparison was performed. Early use was prescribed in CM with MOH sufferers (17 (85.0%)). The primary barrier was cost (19 (90.5%)), followed by a strong agreement with earlier use and region-specific guidance.

Conclusions

Diagnostic delays, high treatment resistance, and significant comorbidity burden characterize CM management in Brazil. In the opinion of the surveyed specialists, anti-CGRP mAbs are perceived as effective and well tolerated, particularly in patients with MOH, and most respondents favored their earlier positioning in the treatment algorithm.

## Introduction

Chronic migraine (CM) is defined as ≥15 headache days per month with at least eight days meeting migraine criteria, and it carries substantially greater disability, socioeconomic burden, and treatment complexity than episodic migraine [1-5]. CM remains underdiagnosed and undertreated: prevalence estimates range from 1.4-2.25% globally [1] to about 2% in Western population-based cohorts [6]; these figures are broadly concordant, and the small numerical difference reflects variation in study populations and case-ascertainment methods rather than a true discrepancy. Diagnosis is further complicated by the gradual, often insidious transition from episodic to CM [6], the high prevalence of medication-overuse headache (MOH), which creates a therapeutic paradox when withdrawal is poorly tolerated [7], and the substantial burden of psychiatric comorbidity, particularly anxiety and depression [6,8]. These overlapping challenges are compounded by high rates of prior preventive treatment failure: discontinuation due to poor tolerability exceeds 60%, and overall discontinuation exceeds 28%, reflecting limited confidence in conventional preventive options [9,10].

The identification of calcitonin gene-related peptide (CGRP) as a central mediator of migraine pathophysiology enabled the development of targeted, mechanism-based therapies, including the anti-CGRP monoclonal antibodies (mAbs) fremanezumab and galcanezumab [11,12]. In randomized controlled trials (RCTs), these agents robustly and reproducibly reduce monthly migraine days, improve quality of life, and achieve high responder rates compared with placebo, with a favorable safety profile and no serious adverse events reported in trial populations [12-14]. This trial-level evidence is distinct from, and should not be conflated with, the expert-opinion data reported in the present survey; the two represent different levels of evidence, and we make this distinction explicit throughout this manuscript.

Despite this evidence base, real-world practice in Brazil and other Latin American health systems is poorly characterized. Real-world patients differ substantially from RCT populations, they more often present with treatment-refractory disease, comorbidities, and prior treatment failures [15] and key clinical questions remain unresolved locally: whether mAbs can be used first-line in selected patients versus only after failure of conventional preventives; how they should be used in patients with concurrent MOH, a population often excluded from trials [16]; whether combination with standard preventives is advisable; and how clinicians should define an adequate treatment trial and response [17]. Access, reimbursement, and prescribing patterns in Brazil's mixed public-private health system add further real-world variability not captured by controlled trial designs [15,18-21], and Latin American populations remain underrepresented in the migraine literature generally [20].

Given this gap, and in the absence of sufficient regional data to support a formal, resource-intensive multi-round consensus process, the Brazilian Academy of Headache and Orofacial Pain elected to conduct a structured cross-sectional survey of its own titular specialists as a pragmatic, exploratory first step toward a position statement. A full Delphi process, involving iterative, anonymous multi-round rating with controlled feedback, was considered but judged premature in the absence of prior descriptive data on regional practice patterns; the present survey is intended to characterize current opinion and practice as groundwork for a future, more formal consensus exercise. The main objective of this survey was to evaluate Brazilian headache specialists' perceptions of the efficacy, safety, and tolerability of fremanezumab and galcanezumab and their views on therapeutic positioning in everyday clinical practice, in order to inform a Brazilian Academy of Headache and Orofacial Pain position statement, with language chosen to align with the framing already used in the Abstract. These findings are intended to support region-specific strategies for CM management in Brazil and Latin America; as an opinion survey, they characterize current expert perception and practice patterns and are not intended to substitute for evidence-based clinical guidelines.

## Materials and methods

Study design

A cross-sectional, expert-opinion survey of 21 Brazilian headache specialists was carried out to inform a position statement of the Brazilian Academy of Headache and Orofacial Pain. The survey (Appendices) was conducted electronically via Google Forms (Google LLC, Mountain View, CA) between March 18 and March 30, 2026. This study was designed to inform a position statement. A single cross-sectional survey, rather than a formal multi-round Delphi consensus process, was chosen because no prior descriptive data existed on regional practice patterns for these agents; the survey was intended as a pragmatic, exploratory step to characterize current opinion and inform, rather than substitute for, a future formal consensus process. This choice is discussed further, together with its limitations, in the Limitations section. This study was designed to inform a position statement, based on expert opinion, and does not report clinical outcomes or effectiveness data.

Expert panel selection

To ensure sufficient clinical experience in the management of CM, purposive sampling was conducted among headache specialists practicing in Brazil. The qualified participants are titular members of the Brazilian Academy of Headache and Orofacial Pain and must be practicing active clinical medicine in the treatment of headache disorders. Meritocracy was the main criterion for including the participants in the present survey. Meritocracy, as used here, refers operationally to two criteria: titular Academy membership and active clinical practice in headache medicine; these together constituted the sole basis for inclusion. The number of specialists invited and the resulting response rate are not available and are noted as a limitation. Informed consent was obtained from all participants before the survey.

Survey development

The survey instrument will include 49 structured questions divided into five themes, which are (1) Clinical Experience With CM (nine questions), (2) Fremanezumab and Galcanezumab in CM (33 questions), (3) Comparison Between Fremanezumab and Galcanezumab (five questions), (4) Patient Selection and Therapeutic Positioning (five questions), and (5) Opinion Consensus (eight questions). The survey was developed based on a literature review and clinical expertise. The instrument was not formally validated and did not undergo structured pilot testing, item reduction, or reliability assessment prior to distribution; this is acknowledged as a methodological limitation. Fremanezumab and galcanezumab are the only two mAbs currently available in Brazil.

The survey used various response formats to elicit participants' perspectives and clinical practices. Demographic and practice characteristics were noted using categorical selections. Efficacy was measured on a Likert scale (0 (ineffective) to 5 (extremely effective)), and safety on a Likert scale (0 (very unsafe) to 4 (very safe)). The frequency scale ranged from 1 (always) to 5 (never or no experience) to measure prescription patterns. The level of agreement with the consensus statements was measured on a five-point Likert scale (1 strongly disagree, 5 strongly agree). Also, a series of multiple-choice questions was added to obtain data about comorbidities and a range of clinical situations. Qualitative responses were also allowed in open-ended text boxes to enable more detailed feedback, especially when the respondent chose Other (please specify). Questions were given in Portuguese to accommodate the Brazilian participant population. The questionnaire was designed to capture both quantitative and qualitative clinical information on the use of anti-CGRP mAbs.

Consensus definition

For consensus analysis, statements with ≥80% agreement (4-5 on the 5-point agreement scale) were considered to have strong consensus. Statements that had 70-79% agreement were considered to represent moderate consensus.

Data collection

Survey responses were collected anonymously, and the option to provide a name was at the respondent's discretion. The electronic system documented the timestamp, content of responses, and status of each submission's completion. Respondents were allowed to edit their answers until they were submitted. No incentives were provided for participation.

Outcome measures

The study evaluated primary and secondary outcomes reflecting clinician perspectives and self-reported practice, not patient-level clinical outcomes. Primary outcomes were efficacy scores for fremanezumab and galcanezumab across four main aspects: reduction in headache days per month, reduction in migraine days, reduction in acute medication use, and reduction in disability and quality of life. Furthermore, it evaluated the safety and tolerability profiles of both mAbs and compared their effectiveness.

Secondary outcomes focused on broader clinical and practice-related aspects. These included patterns of clinical practice, such as the volume of patients with CM, the prevalence of the condition, diagnostic accuracy, the time taken to diagnose a patient with the condition correctly, and the prevalence of MOH. The survey also addressed patterns of treatment resistance, defined as failure of two or more preventive medications, and identified barriers to mAbs utilization. In addition, it evaluated therapeutic positioning choices, such as line of treatment, dosage regimen preferences for fremanezumab, and the length of the treatment trial before accepting therapeutic failure. Other aspects of the decision-making between agents were also studied, including the degree of agreement with position statements on the management of CMs and the use of anti-CGRP therapies.

Data analysis

Descriptive statistics were calculated for all survey responses. Frequencies and percentages were used to report categorical variables. In the case of Likert scale responses, the distribution across the points on the scale was given.

Consensus was defined as above (≥80% strong, 70-79% moderate). Comparative questions and individual efficacy ratings for fremanezumab and galcanezumab were analyzed descriptively; no inferential statistical testing was performed given the exploratory nature of this position-statement survey and the small sample size (n = 21), and comparative statements in the Results and Discussion should accordingly be read as descriptive observations rather than statistically confirmed differences.

Missing data were addressed using available-case analysis, with denominators adjusted to the number of responses received for each item; because not all respondents answered every question, denominators (n) are reported explicitly alongside every percentage in the tables below, and readers should refer to the stated denominator rather than assume n = 21 throughout.

Qualitative responses to open-ended items were reviewed for thematic content but were not subjected to formal qualitative coding, given their limited volume and context-specific nature.

Results are reported as percentages accompanied by absolute frequencies (n). Given the small sample size, all percentages should be interpreted as point estimates with correspondingly wide confidence intervals; for example, the reported 81.0% experience rate with fremanezumab (17/21) corresponds to an approximate 95% Wilson confidence interval of 60.0-92.3%. Item-by-item confidence intervals are not displayed in the tables for space reasons but are available from the authors on request. Findings were used to generate consensus-based observations, which are presented as expert opinion and should not be interpreted as evidence-based clinical recommendations.

Ethical considerations

This study used a survey methodology to collect professional opinions from healthcare specialists regarding established therapeutic interventions. Participation was voluntary, and completion of the electronic survey was considered to indicate informed consent. No patient-level data, identifiable personal information, or clinical records were collected. In accordance with Brazilian National Health Council (Conselho Nacional de Saúde (CNS)) Resolution No. 510/2016, research consisting exclusively of anonymous professional opinion surveys that do not involve patients or identifiable personal data and present no risks beyond those encountered in everyday life is exempt from mandatory review by the Research Ethics Committee/National Research Ethics Commission (CEP/CONEP) system. 

## Results

Participant characteristics

Twenty-one headache specialists participated in the survey. The majority of respondents were neurologists (18 (85.7%)), and most had a long clinical history, with 14 (66.7%) having over 20 years of experience treating headache disorders (Table 1). There was a wide range of practices regarding monthly CM patient volume. This difference reflects the different practice environments used in the survey.

**Table 1:** Participant demographics and chronic migraine practice characteristics Note: The “Few reports” category (n = 1) does not correspond to a numeric volume bin and instead reflects a free-text/qualitative response provided by one respondent rather than a discrete quantitative category; it is retained here as reported and should not be treated as directly comparable to the numeric bins above.

Characteristic	n	%
Years treating headache disorders		
Less than 5 years	1	4.80
5-10 years	2	9.50
11-20 years	4	19.00
More than 20 years	14	66.70
Primary specialty		
Neurology	18	85.70
Other (orofacial pain, neuroscience)	3	14.30
Monthly chronic migraine patient volume		
Few reports	1	4.76
<10 patients	5	23.80
10-30 patients	7	33.30
31-60 patients	2	9.52
61-80 patients	3	14.30
120 patients	1	4.76
200 patients	2	9.52

Burden and clinical challenges of CM

Clinical Experience With CM

**Figure 1:**
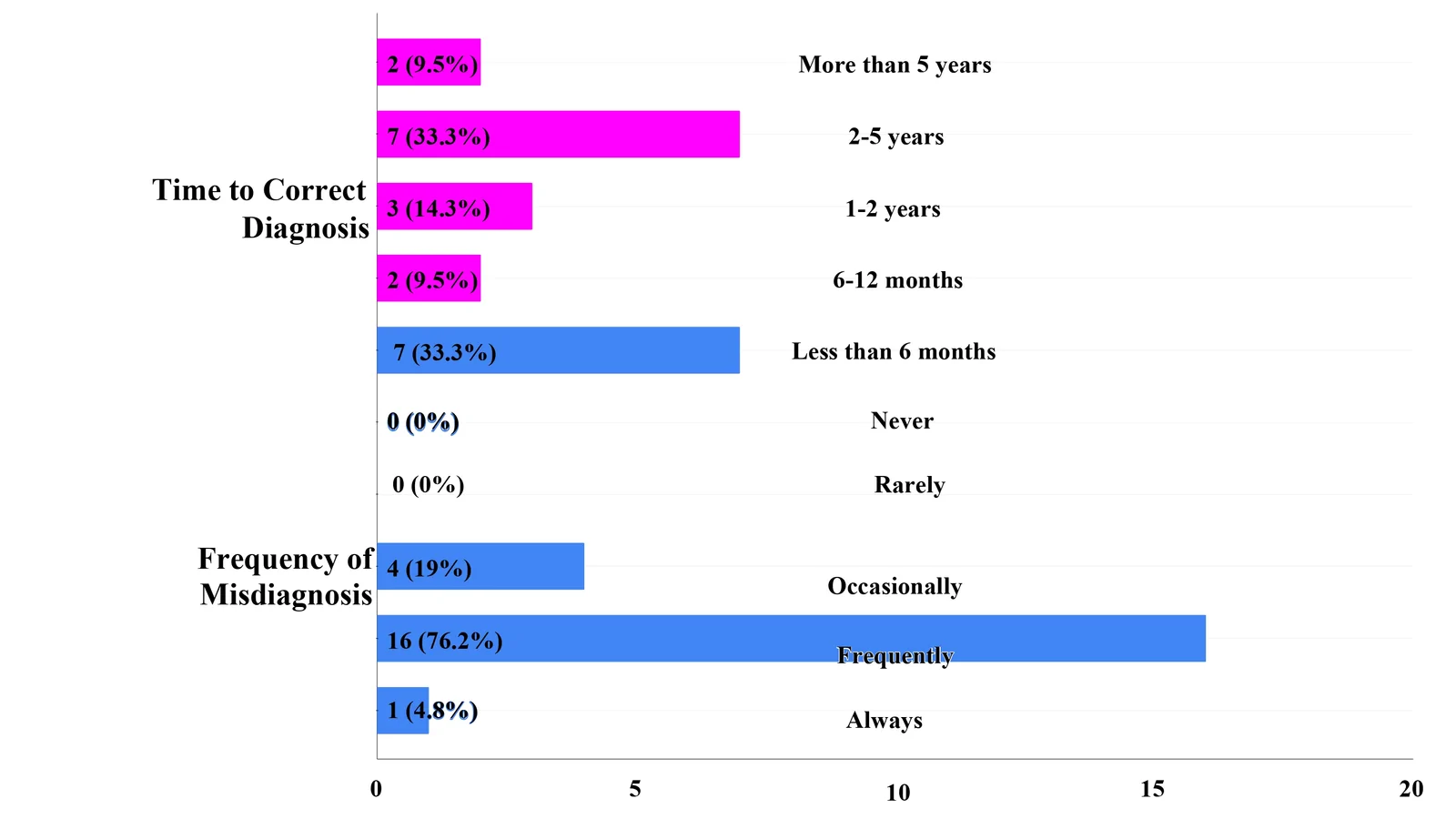
Frequency of misdiagnosis and time to correct diagnosis

Misdiagnosis at initial presentations was common. The majority of clinicians stated that CM patients presented with a false diagnosis, with a frequency of frequently (16 (76.2%)) or always (one (4.8%)), and none of them replied with rarely or never. The time to correct diagnosis differed significantly, with 12 (57.1%) of participants reporting that it usually took one year or more to clarify the diagnosis, and a third reporting a delay of two to five years (Figure 1).

**Figure 2:**
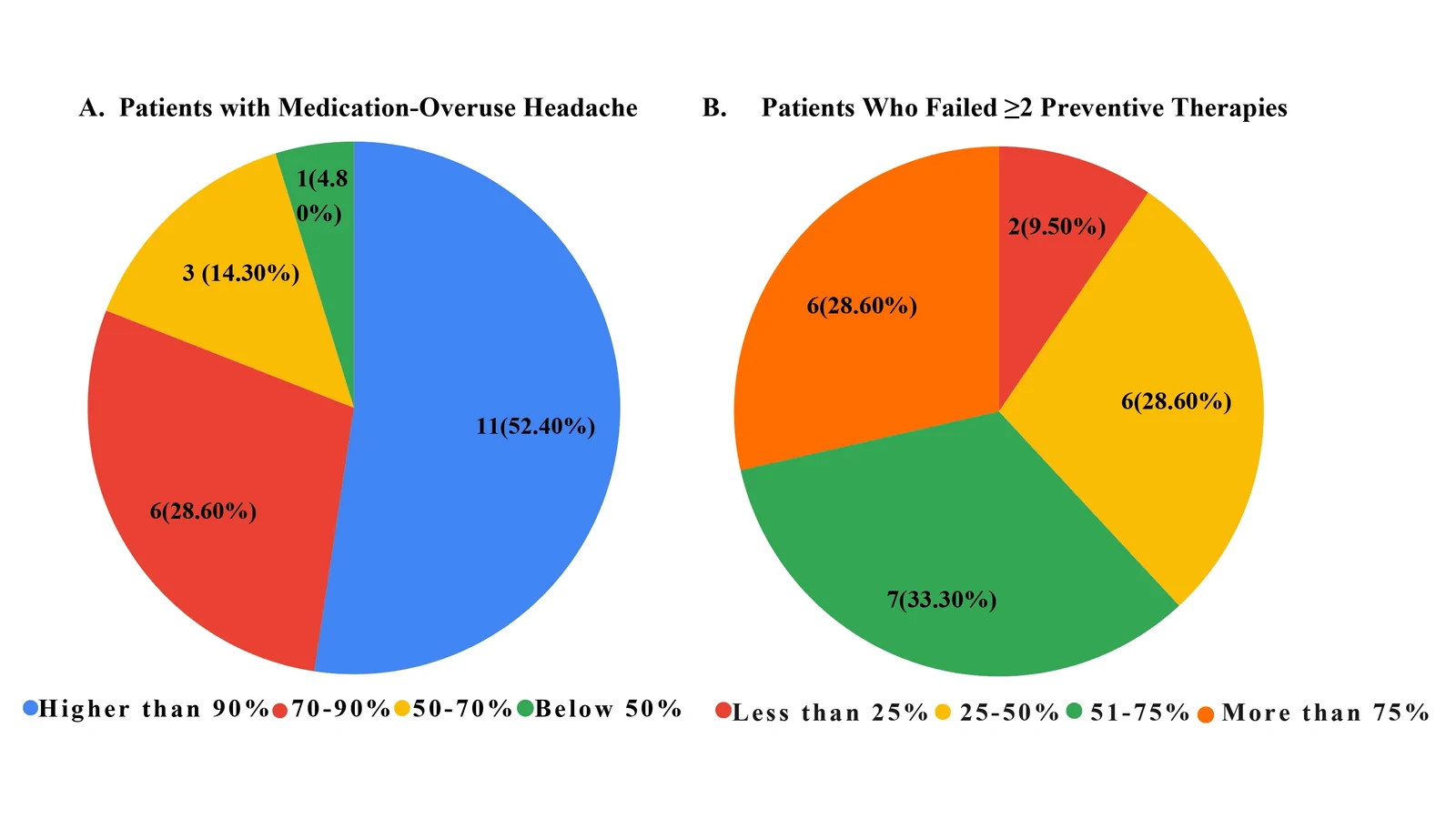
(A) Patients with medication-overuse headache and (B) patients who failed ≥2 preventive therapies

Treatment resistance was frequently reported, with almost two-thirds of clinicians (13 (61.9%)) suggesting that over half of their CM patients had not responded to two or more preventive treatments. MOH was very common, with 11 (52.4%) of the respondents estimating that more than 90% of their CM patients had concurrent MOH (Figure 2).

Comorbidity burden was substantial. Most often reported comorbidities affecting CM management were psychiatric (16 (76.2%)), sleep-related (15 (71.4%)), and anxiety (13 (61.9%)). Comorbidities like musculoskeletal and metabolic conditions, such as fibromyalgia (eight (38.1%)) and obesity (seven (33.3)), were also prevalent (Table 2).

**Table 2:** Comorbidities most impacting chronic migraine management (n = 21)

Comorbidity	Number of Responses (n)	Percentage (%)
Anxiety	16	76.20
Depression	15	71.40
Insomnia	13	61.90
Fibromyalgia	8	38.10
Obesity	7	33.30
Neck pain	5	23.80
Temporomandibular disorder	3	14.30
Obstructive sleep apnea	3	14.30
Other	0	0

Clinical use and experience with anti-CGRP mAbs

Utilization Patterns

The use of anti-CGRP mAbs was also experienced with 17 (81.0%) and 18 (85.7%) of the respondents using fremanezumab and galcanezumab, respectively (Table 3). Among respondents with experience, prescription frequency ranged from occasional to frequent for both agents; only a minority reported using either agent routinely (“always”) in their practice.

**Table 3:** Clinical experience with anti-CGRP monoclonal antibodies Note: Denominators vary slightly across rows for fremanezumab (n = 17 “Experience,” rising to n = 18 for subsequent efficacy/safety/tolerability items) and galcanezumab (n = 18 “Experience,” rising to n = 19 for Prescription Frequency and subsequent items). This reflects one additional respondent per agent who had not personally prescribed the medication but nonetheless provided a rating based on observed patient outcomes or consultation experience; these denominator shifts are a limitation of the survey's skip-logic design and are noted here for transparency. ᵃRespondents selected the single most commonly observed adverse event; percentages were calculated using valid responses for fremanezumab (n = 17) and galcanezumab (n = 19). ᵇOther represents additional adverse events reported by respondents.

Variable	Category	Fremanezumab n (%)	Galcanezumab n (%)
Experience with medication	Yes	17 (81.0)	18 (85.7)
	No	4 (19.0)	3 (14.3)
Prescription frequency	-	n = 17	n = 19
	Always	1 (5.9)	2 (10.5)
	Frequently	6 (35.3)	6 (31.6)
	Occasionally	8 (47.1)	9 (47.4)
	Rarely	2 (11.8)	1 (5.3)
	Never/no experience	0 (0.0)	1 (5.3)
Efficacy: reducing monthly headache days	-	n = 18	n = 20
	Ineffective	1 (5.9)	1 (5.0)
	Slightly effective	0 (0.0)	0 (0.0)
	Moderately effective	0 (0.0)	3 (15.0)
	Effective	6 (35.3)	4 (20.0)
	Very effective	9 (50.0)	8 (40.0)
	Extremely effective	2 (11.8)	4 (20.0)
Efficacy: reducing migraine days	-	n = 18	n = 20
	Ineffective	1 (5.6)	1 (5.0)
	Slightly effective	0 (0.0)	0 (0.0)
	Moderately effective	0 (0.0)	3 (15.0)
	Effective	5 (27.8)	2 (10.0)
	Very effective	9 (50.0)	9 (45.0)
	Extremely effective	3 (16.7)	5 (25.0)
Effect on acute medication use	-	n = 18	n = 20
	Ineffective	1 (5.6)	1 (5.0)
	Slightly effective	0 (0.0)	0 (0.0)
	Moderately effective	2 (11.1)	1 (5.0)
	Effective	8 (44.4)	4 (20.0)
	Very effective	4 (22.2)	6 (30.0)
	Extremely effective	3 (16.7)	8 (40.0)
Impact on disability/quality of life	-	n = 18	n = 20
	Ineffective	1 (5.6)	1 (5.0)
	Slightly effective	1 (5.6)	0 (0.0)
	Moderately effective	0 (0.0)	2 (10.0)
	Effective	6 (33.3)	3 (15.0)
	Very effective	6 (33.3)	8 (40.0)
	Extremely effective	4 (22.2)	6 (30.0)
Safety rating	-	n = 18	n = 19
	Very unsafe	1 (5.6)	1 (5.3)
	Slightly safe	0 (0.0)	0 (0.0)
	Moderately safe	0 (0.0)	2 (10.5)
	Safe	3 (16.7)	1 (5.3)
	Very safe	14 (77.8)	15 (78.9)
Tolerability rating	-	n = 18	n = 19
	Very unsafe	1 (5.6)	1 (5.3)
	Slightly safe	0 (0.0)	0 (0.0)
	Moderately safe	1 (5.6)	2 (10.5)
	Safe	1 (5.6)	3 (15.8)
	Very safe	15 (83.3)	13 (68.4)
Most common adverse eventsᵃ	-	n = 17	n = 19
	Injection-site reaction	12 (70.6)	14 (73.7)
	Constipation	2 (11.8)	0 (0.0)
	Fatigue	1 (5.9)	1 (5.3)
	Post-injection headache	0 (0.0)	0 (0.0)
	No relevant adverse events	1 (5.9)	3 (15.8)
	Otherᵇ	1 (5.9)	1 (5.3)
Therapeutic positioning	-	n = 19	n = 20
	First-line in selected cases	12 (63.2)	13 (65.0)
	After the failure of 1 preventive	2 (10.5)	2 (10.0)
	After the failure of 2 preventives	3 (15.8)	4 (20.0)
	After failure of 3+ preventives	1 (5.3)	0 (0.0)
	Only after botulinum toxin failure	0 (0.0)	0 (0.0)
	Should not be routinely used	1 (5.3)	1 (5.0)
Preferred dosing regimen	-	n = 19	-
	Monthly	11 (57.9)	-
	Quarterly	1 (5.3)	-
	Depends on patient profile	6 (31.6)	-
	Not enough experience	1 (5.3)	-

Efficacy Outcomes

Respondents rated both mAbs as effective across the surveyed clinical domains, reflecting clinician perception rather than measured patient outcomes. Fremanezumab and galcanezumab were rated very-to-extremely effective for reducing migraine days by roughly two-thirds of respondents, with similar patterns for headache days per month and disability/quality-of-life improvement. Galcanezumab received numerically higher ratings for reduction in acute medication use and for overall patient-reported outcomes than fremanezumab; because no statistical comparison was performed, this numerical difference should not be interpreted as a demonstrated difference in clinical effectiveness between the two agents (Table 3).

Safety and Tolerability Profile

Both agents were rated favorably for safety and tolerability by responding clinicians. Most of the respondents indicated that fremanezumab (14 (77.8)) and galcanezumab (15 (78.9)) were very safe. Tolerability ratings were also high. The adverse events most frequently reported with both treatments were injection-site reactions, and other adverse events were very rare (Table 3).

Comparison between fremanezumab and galcanezumab

**Figure 3:**
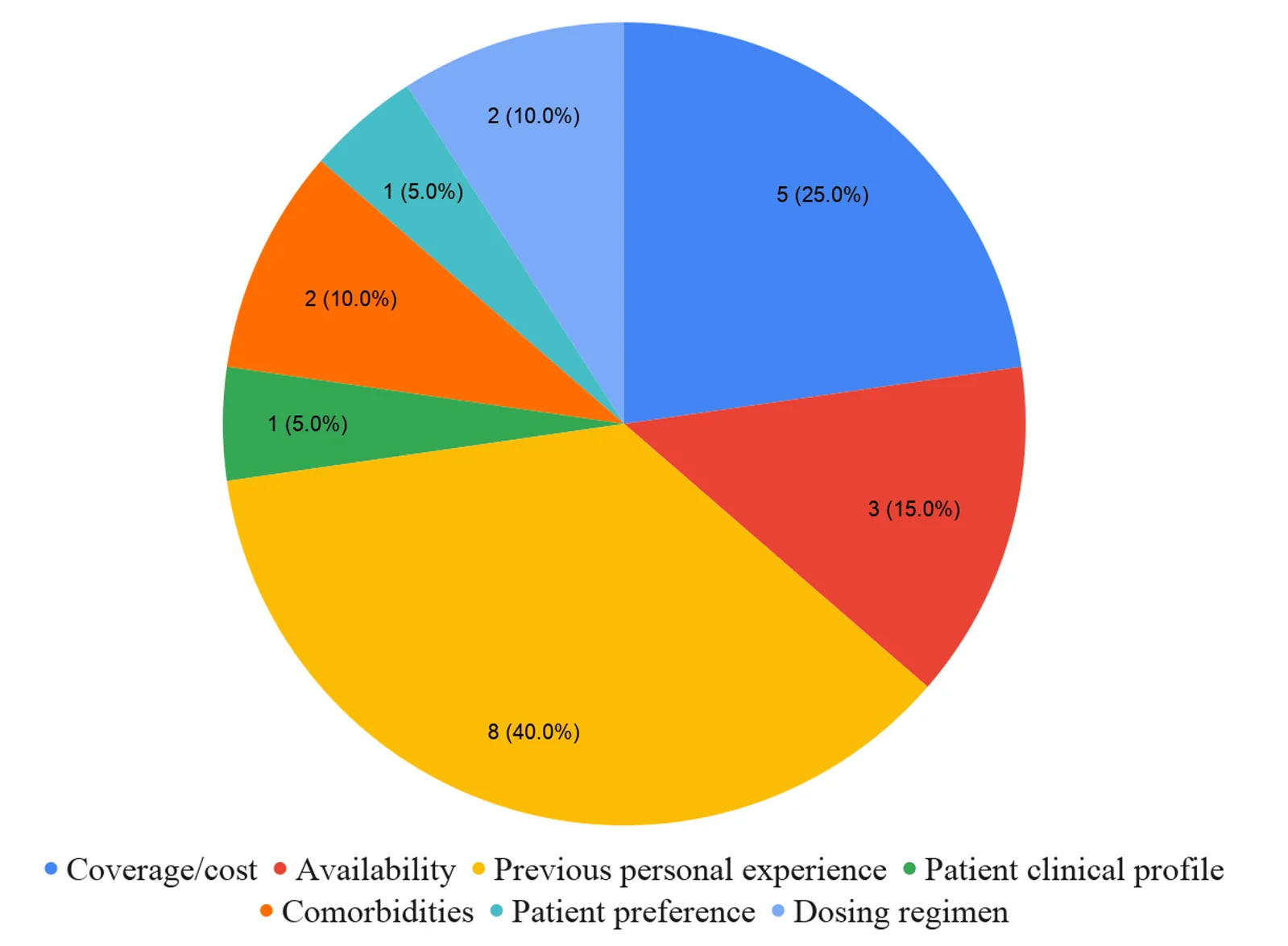
Factors influencing choice between fremanezumab and galcanezumab

Most clinicians perceived no notable difference in efficacy or tolerability between fremanezumab and galcanezumab (Table 4); as noted above, these comparative perceptions were not evaluated with statistical testing. For overall CM efficacy, 11 (52.4%) reported no difference, with smaller proportions favoring galcanezumab (four (19.0%)) or fremanezumab (three (14.3%)); a similar pattern was seen for tolerability. Responses were more distributed for speed of onset and adherence, with galcanezumab showing a numerical, statistically untested advantage in both (Table 4). When choosing between agents, previous clinical experience (eight (40.0%)) and cost/coverage considerations (five (25.0%)) were the most commonly cited influences; dosing preferences were cited less often (Figure 3).

**Table 4:** Comparative assessments

Comparison Metric	Fremanezumab	Galcanezumab	No Relevant Difference	Insufficient Experience
Overall efficacy for chronic migraine	3 (14.3%)	4 (19.0%)	11 (52.4%)	3 (14.3%)
Tolerability	2 (9.5%)	5 (23.8%)	11 (52.4%)	3 (14.3%)
Faster onset of response	1 (4.8%)	7 (33.3%)	10 (47.6%)	3 (14.3%)
Better patient adherence	4 (19.0%)	7 (33.3%)	8 (38.1%)	2 (9.5%)

When selecting between agents, previous clinical experience (eight (40.0%)) and cost or coverage considerations (five (25.0%)) were the most influential factors. Dosing preferences were less frequently cited (Figure 3).

Treatment patterns, patient selection, and therapeutic positioning

Initiation Criteria

Common indications for initiating anti-CGRP mAbs included intolerance to oral preventive therapies (13 (65.0%)), high functional disability (13 (65.0%)), and failure of ≥2 preventive treatments (12 (60.0%)). Other factors included contraindications to conventional preventives and medication overuse (Table 5).

**Table 5:** Patient selection and therapeutic positioning for anti-CGRP monoclonal antibodies *Respondents could select more than one indication; therefore, percentages do not sum to 100%.

Domain	Survey Item	Response Category	n (%)
Indications for initiation*	Failure of ≥2 preventive treatments	Selected	12 (60%)
	Intolerance to oral preventive treatments	Selected	13 (65%)
	Contraindication to traditional preventives	Selected	10 (50%)
	High functional disability	Selected	13 (65%)
	Medication overuse	Selected	10 (50%)
	Failure of botulinum toxin	Selected	4 (20%)
	Informed patient preference	Selected	9 (45%)
Use in medication overuse	Fremanezumab/galcanezumab is appropriate in chronic migraine with MOH	Yes, frequently	17 (85%)
		Yes, in selected cases	2 (10%)
		Only after prior detoxification	0 (0%)
		No	1 (5%)
Combination therapy at initiation	Combine other preventives with mAb at the start	Yes, usually	10 (50%)
		Yes, in selected cases	6 (30%)
		No, prefer monotherapy	3 (15%)
		Depends on the case	1 (5%)
Time to assess treatment failure	When is mAb considered failed in chronic migraine	3 months	8 (40%)
		6 months	9 (45%)
		Depends on the case	3 (15%)
		1 month	0 (0%)
		2 months	0 (0%)
Criteria for adequate response	Key determinant of clinical response	Combination of factors	16 (80%)
		Reduction in headache/migraine days	2 (10%)
		Reduction in disability	1 (5%)
		Global patient-reported improvement	1 (5%)
		Reduction in acute medication use	0 (0%)

Therapeutic Positioning

About two-thirds of respondents favored early use of fremanezumab and galcanezumab as first-line therapy in selected cases (Table 3); this figure describes the proportion of surveyed specialists endorsing this option, not a validated first-line indication or a finding of comparative clinical superiority.

Treatment Patterns and Response Assessment

Most respondents (17 (85.0%)) considered anti-CGRP mAbs suitable for patients with CM and co-occurring MOH without requiring prior detoxification, and a further two (10.0%) favored use in selected cases; these are clinician opinions and were not validated against patient-level treatment outcomes. Duration allowed to judge treatment failure varied: eight (40.0%) of clinicians assessed failure within three months and nine (45.0%) within six months. Most respondents (16 (80.0%)) reported using a combination of outcome measures rather than a single measure to judge response. Combination therapy at initiation was common, with 10 (50.0%) reporting regular use and six (30.0%) selective use alongside anti-CGRP mAbs (Table 5).

Barriers to clinical implementation

Economic factors emerged as the primary barrier to the utilization of anti-CGRP mAbs. The most common barrier cited by clinicians was cost (19 (90.5%)). Compared with conventional barriers to implementation, such as health system/insurance coverage of procedures, regulatory approval standards, physician acquaintance, patient resistance, and drug availability, none of the respondents noted these as major challenges (0% across all categories). Two clinicians (9.5%) noted other barriers related to prescribing authority patterns and referral systems within their healthcare facilities.

Consensus on regional management priorities

There was a high degree of consensus between major statements (Table 6). Nearly all respondents (100%) strongly agreed that cost and coverage are the main obstacles to accessing anti-CGRP therapy. High agreement was also reported regarding the role of MOH as a management challenge and the underdiagnosis of CM in the region. Both fremanezumab and galcanezumab were widely endorsed as options for difficult-to-treat CM, and most respondents favored earlier positioning of these agents in the treatment algorithm. Most respondents also agreed that access-related factors influence agent choice more than perceived clinical differences and that region-specific consensus guidance is needed. As with all findings in this survey, these consensus statements reflect the aggregated opinion of 21 specialists and should be interpreted as expert consensus rather than as evidence of clinical superiority or effectiveness. 

**Table 6:** Consensus statement analysis Agreement scale: 1 = strongly disagree, 2 = partially disagree, 3 = neutral/uncertain, 4 = partially agree, 5 = strongly agree

Statement	1	2	3	4	5
Chronic migraine remains underdiagnosed in many Latin American countries	2 (9.5%)	0 (0%)	0 (0%)	4 (19.0%)	15 (71.4%)
Medication overuse is an important barrier in chronic migraine management	1 (5.0%)	0 (0%)	1 (5.0%)	3 (15.0%)	15 (75.0%)
Fremanezumab is an effective option for difficult-to-treat chronic migraine	0 (0%)	0 (0%)	3 (16.7%)	3 (16.7%)	12 (66.7%)
Galcanezumab is an effective option for difficult-to-treat chronic migraine	0 (0%)	0 (0%)	1 (5.3%)	5 (26.3%)	13 (68.4%)
Anti-CGRP therapies should be considered earlier in the therapeutic algorithm	1 (5.3%)	0 (0%)	1 (5.3%)	6 (31.6%)	11 (57.9%)
Cost and coverage remain the main limiting factors for fremanezumab and galcanezumab use	0 (0%)	0 (0%)	0 (0%)	0 (0%)	19 (100%)
The choice between fremanezumab and galcanezumab depends more on access-related factors than clinical differences	0 (0%)	2 (10.5%)	1 (5.3%)	2 (10.5%)	14 (73.7%)
Need for region-specific consensus recommendations for Latin America	0 (0%)	1 (5.3%)	1 (5.3%)	4 (21.1%)	13 (68.4%)

## Discussion

This position statement offers clinician-reported, opinion-based insight into CM management in Brazil and Latin America, highlighting diagnostic difficulty, high disease burden, and a reported clinical preference toward anti-CGRP mAbs. These findings describe a mismatch between international evidence-based guidance and self-reported local practice as perceived by the surveyed specialists; because the study is a descriptive opinion survey rather than a comparative or outcomes study, the findings should be read as hypothesis-generating rather than as demonstrated practice-guideline evidence.

A key observation from this study is the presence of diagnostic delays and misclassification in CM, consistent with the existing literature. Almost half of migraine patients are misdiagnosed initially, usually with tension-type headache or sinusitis, which demonstrates significant diagnostic gaps during initial clinical interactions [22]. Similarly, delayed or inappropriate diagnosis has been observed in up to 65% of cases, especially when the patients are initially assessed by non-experts, causing a long-term disease burden and higher healthcare expenditures [23]. The dynamic and progressive characteristics of migraine further complicate the identification, with the changing patterns of symptoms [24]. Additionally, CM is often misdiagnosed, and only a small proportion of patients are correctly diagnosed despite the set criteria [25]. The coexistence of MOH further complicates the situation. It is both similar to migraine and worsens it by postponing the proper treatment [26]. These findings state the need for improved clinician awareness and early diagnostic strategies.

The present findings highlight the high costs of treatment resistance in CM, which is in line with real-world data of the BECOME study, where a large percentage of patients reported previous preventive treatment failures and high disability [27]. This highlights the continued weakness of traditional preventative treatment, such as poor effectiveness and safety. Moreover, the presence of psychiatric and sleep-related comorbidities observed supports their importance in the development of the severity of the disease and its treatment. Previous studies show that anxiety and depression are closely related to the frequency of migraine, disability, and worse quality of life [28]. Also, the complexity of resistant and refractory migraine highlights the necessity of personalized, multimodal approaches to treatment that can address both biological and psychosocial aspects of care [29].

Respondents' perceptions of fremanezumab and galcanezumab efficacy are directionally consistent with published trial and real-world data showing reductions in migraine frequency and disability and improved quality of life [30,31], and the favorable safety/tolerability ratings reported here echo published long-term safety data [32,33]. However, this survey did not measure patient outcomes directly, and these parallels should be read as context for, rather than confirmation of, the clinicians' perceptions reported here. The largely similar effectiveness ratings across agents are consistent with reports that anti-CGRP mAbs are broadly substitutable in clinical use [34]. Despite these favorable perceptions, use remains conservative, and cost was the most frequently cited constraint, consistent with broader evidence of underutilization of anti-CGRP therapies relative to their trial-demonstrated efficacy, underscoring the need for improved affordability and access [31].

A notable finding of this survey is the preference expressed by respondents for earlier positioning of anti-CGRP mAbs in the treatment algorithm. This preference is directionally aligned with emerging literature suggesting these mechanism-based, well-tolerated agents may be appropriate for first-line use in selected patients [35,36]; it is also broadly consistent with recent international guidance from bodies, such as the American Headache Society and European Headache Federation, which have similarly moved toward earlier positioning in defined patient subgroups. Although the specific eligibility criteria proposed internationally are more granular than those captured in this survey and not all guideline bodies concur on unrestricted first-line use, this is a divergence worth noting explicitly rather than eliding. It should be emphasized, however, that in the present survey this reflects the opinion of 21 Brazilian specialists that mAbs may be appropriate first-line “in selected cases,” not a validated indication change, a comparative-efficacy finding, or a recommendation grounded in outcome data.

The consensus favoring use of anti-CGRP mAbs in patients with MOH without requiring prior detoxification also reflects a perceived shift in management, as emerging evidence suggests that the effectiveness of these agents may not depend on prior medication withdrawal [37]. This remains a debated area: other authors have argued that discontinuing the overused acute medication continues to be an important component of MOH management even when mAbs are used [37], and the present consensus should be read as one clinical viewpoint among several rather than a settled recommendation. The minimal perceived difference between fremanezumab and galcanezumab reported here is consistent with head-to-head and observational studies reporting broadly similar efficacy and safety profiles [38], reinforcing that access and cost, rather than demonstrated clinical superiority, may often drive agent selection in this survey's respondents.

Finally, the strong consensus on region-specific guidelines highlights a persistent evidence gap in Latin America. Underrepresentation in clinical research limits external validity and delays the adoption of locally applicable data into practice, which strengthens the need to rely on extrapolated evidence [39]. This is especially important as migraine has a high burden and heterogeneous presentation, and there are gaps in diagnosis and access to treatment throughout the region [40,41]. Thus, it is necessary to enhance research capacity in the region to inform equal, context-sensitive approaches to CM management [39].

In summary, this survey identifies clinician-perceived gaps in CM care in Brazil: diagnostic delay, high treatment resistance, and limited access to mAb therapy, which are broadly consistent with the international literature. Because these findings derive from a small, self-selected sample of specialists reporting their own perceptions rather than from patient-level outcome data, they should be understood as an expert-opinion position statement intended to prompt further, more rigorous regional research (including prospective real-world outcome studies and, potentially, a formal Delphi consensus process), rather than as a source of evidence-based practice recommendations in their own right.

Limitations

This study has several limitations. It was based on a small, purposively sampled group of 21 Brazilian headache specialists selected on the basis of Academy membership and active clinical practice; the number of specialists invited and the resulting response rate could not be determined, and the sampling approach may be subject to selection bias favoring clinicians with a particular interest in, or favorable experience of, anti-CGRP mAbs. The survey relied on self-reported clinician perceptions and clinical experience rather than patient-level outcomes, introducing potential recall bias (particularly for retrospective estimates of patient volumes and comorbidity prevalence) and social-desirability or response bias; nonresponse bias cannot be excluded, since item-level denominators varied and not all respondents answered every question. Its cross-sectional design precludes causal inference, and, as an opinion-based descriptive survey, it cannot establish clinical efficacy, safety, or comparative superiority-conclusions on these points would require prospective, patient-level, or comparative outcome data. The survey instrument itself was not formally validated and did not undergo pilot testing or reliability assessment prior to use, which may affect the precision and reproducibility of individual item responses. A formal Delphi or other structured multi-round consensus methodology, involving iterative anonymous rating and controlled feedback, was not used; a single cross-sectional survey provides a weaker basis for consensus-based recommendations than such methods, and this represents an important methodological limitation of the present position statement. Finally, only experts from Brazil participated, and findings may not fully reflect practice patterns, access barriers, or healthcare-system characteristics elsewhere in Latin America. Taken together, these limitations mean that expert opinion of the kind reported here occupies a lower level of evidence than comparative clinical or real-world outcome studies, and the recommendations that follow should be weighted accordingly.

## Conclusions

This position statement identifies clinician-perceived gaps in the care of CM in Brazil and Latin America, including diagnostic delay, high treatment-resistance rates, and substantial comorbidity burden. In the opinion of the surveyed specialists, anti-CGRP mAbs are considered effective and safe in real-world practice, and may be appropriate for earlier use in the treatment algorithm, particularly among patients with difficult-to-treat migraine and MOH; however, these are expert-consensus observations derived from clinician-reported perceptions, not conclusions established by comparative or patient-level outcome data, and should be interpreted and applied as such. Access to these agents nonetheless remains constrained by cost. These findings underscore the need for improved diagnostic pathways, more equitable access to advanced therapies, and multidisciplinary care, and they highlight specific priorities for future regional research, including prospective outcome studies and a formal consensus process, that would allow these opinion-based observations to be tested and, where appropriate, translated into evidence-based, context-specific guidelines for CM management in the region.

## Appendices

Analysis of data from Google Forms

Chronic Migraine and Mabs

Section 1. Clinical Experience With Chronic Migraine

1. How many years have you treated headache disorders?

2. What is your primary specialty?

3. What percent of your patients have chronic migraine?

4. How many chronic migraine patients do you see monthly?

5. How often do chronic migraine patients arrive misdiagnosed?

6. What is the average time to correct a chronic migraine diagnosis?

7. What percent of chronic migraine patients have medication-overuse headache?

8. Which comorbidities most impact chronic migraine management?

9. What percent of your patients failed ≥2 preventives?

Section 2. Fremanezumab and Galcanezumab in Chronic Migraine

Scales for the following questions:

-        Efficacy scale: 0 = Ineffective, 1 = Slightly effective, 2 = Moderately effective, 3 = Effective, 4 = Very effective, 5 = Extremely effective

-        Safety scale: 0 = Very unsafe, 1 = Slightly safe, 2 = Moderately safe, 3 = Safe, 4 = Very safe

-        Prescription frequency: 1 = Always, 2 = Frequently, 3 = Occasionally, 4 = Rarely, 5 = Never/no experience

General experience

10. Do you have experience using fremanezumab in chronic migraine?

11. Do you have experience using galcanezumab in chronic migraine?

12. What is the main barrier to using mAbs in chronic migraine?

Specify “others”:

-  “I don't prescribe; when I make a probable diagnosis, I refer the patient to a headache specialist.”

-  “Some patients already come with medication prescribed by a neurologist.”

Fremanezumab

13. How often do you prescribe fremanezumab?

14. How effective is fremanezumab in reducing monthly headache days?

15. How effective is fremanezumab in reducing migraine days?

16. How does fremanezumab affect acute medication use?

17. How does fremanezumab impact disability/quality of life?

18. How would you rate fremanezumab's safety?

19. How would you classify the tolerability of fremanezumab?

20. What adverse events are most common with fremanezumab?

Specify “others”: “Discreto aumento da pressão arterial.”

21. In your opinion, fremanezumab should be considered:

22. Which fremanezumab dosing regimen do you prefer?

Galcanezumab

23. How often do you prescribe galcanezumab?

24. How effective is galcanezumab in reducing headache days?

25. How effective is galcanezumab in reducing migraine days?

26. How does galcanezumab affect acute medication use?

27. How does galcanezumab impact disability/quality of life?

28. How would you rate galcanezumab's safety?

29. How would you rate galcanezumab's tolerability?

30. What adverse events are most common with galcanezumab?

31. In your opinion, galcanezumab should be considered:

Section 3. Comparison Between Fremanezumab and Galcanezumab

32. Which mAb shows better overall efficacy for chronic migraine?

33. Which shows better tolerability?

34. Which has a faster onset of response?

35. Which favors better patient adherence in your practice?

36. What most influences your choice between fremanezumab and galcanezumab?

Section 4. Patient Selection and Therapeutic Positioning

37. When is it most appropriate to start anti-CGRP treatment?

38. Is fremanezumab or galcanezumab appropriate for chronic migraine with medication overuse?

39. Is it appropriate to combine other preventives with an mAb at treatment start?

40. After how long do you judge a mAb as failed in chronic migraine?

41. What is the key criterion for adequate clinical response?

Section 5. Opinion Consensus

For the statements below, please indicate your degree of agreement:

-        1 = Strongly disagree

-        2 = Partially disagree

-        3 = Neutral/uncertain

-        4 = Partially agree

-        5 = Strongly agree

42. Chronic migraine remains underdiagnosed in many Latin American countries.

43. Medication overuse is an important barrier in the management of chronic migraine.

44. Fremanezumab is an effective option for patients with difficult-to-treat chronic migraine.

45. Galcanezumab is an effective option for patients with difficult-to-treat chronic migraine.

46. Anti-CGRP therapies should be considered earlier in the therapeutic algorithm for chronic migraine.

47. Cost and coverage remain the main limiting factors for the use of fremanezumab and galcanezumab in clinical practice.

48. The choice between fremanezumab and galcanezumab usually depends more on access-related factors than on robust clinical differences.

49. There is a need for region-specific consensus recommendations to guide the use of these monoclonal antibodies in Latin America.
